# Philadelphia College of Pharmacy

**Published:** 1861-05

**Authors:** 


PHILADELPHIA COLLEGE OF PHARMACY.—This old institution continues to be in a highly prosperous condition, the class of the last session being 121. Of these, 41 received the degree of "Graduate in Pharmacy." There were 5 graduates at the Maryland College of Pharmacy.

